# Second harmonic generation nanoparticles enables Near-Infrared Photodynamic Therapy from visible light reactive photosensitizer conjugates

**DOI:** 10.1371/journal.pone.0274954

**Published:** 2022-09-29

**Authors:** Ayan Barbora, Fares Yazbak, Svetlana Lyssenko, Vadim Nave, Faina Nakonechny, Paul Ben Ishai, Refael Minnes

**Affiliations:** 1 Faculty of Natural Sciences, Department of Physics, Ariel University, Ariel, Israel; 2 Faculty of Engineering, Department of Chemical Engineering, Ariel University, Ariel, Israel; Harvard Medical School, UNITED STATES

## Abstract

Combination of photosensitizers (PS) with nanotechnology can improve the therapeutic efficiency of clinical Photodynamic Therapy (PDT) by converting visible light reactive PSs into Near-Infrared (NIR) light responsive molecules using Harmonic Nanoparticles (HNP). To test the PDT efficiency of HNP-PS conjugates, pathogenic *S*. *aureus* cell cultures were treated with perovskite (Barium Titanate) Second Harmonic Generation (SHG) nanoparticles conjugated to photosensitizers (PS) (we compared both FDA approved Protoporphyrin IX and Curcumin) and subjected to a femtosecond pulsed Near-Infrared (NIR) laser (800 nm, 232–228 mW, 12–15 fs pulse width at repetition rate of 76.9 MHz) for 10 minutes each. NIR PDT using Barium Titanate (BT) conjugated with Protoporphyrin IX as HNP-PS conjugate reduced the viability of *S*. *aureus* cells by 77.3 ± 9.7% while BT conjugated with Curcumin did not elicit any significant effect. Conventional PSs reactive only to visible spectrum light coupled with SHG nanoparticles enables the use of higher tissue penetrating NIR light to generate an efficient photodynamic effect, thereby overcoming low light penetration and tissue specificity of conventional visible light PDT treatments.

## 1. Introduction

Photodynamic therapy (PDT) uses light, and a chemical substance called a photosensitizer (PS) to generate molecular oxygen and elicit cell death. This phenomenon is called phototoxicity. This promising approach has been in development for various applications including microbial inactivation and the treatment of infections [1]. PDT as a treatment procedure is advantageous over conventional therapies as it provides dual specificity: 1) PSs are targeted to a specific location of cells or tissues and 2) irradiation regimes can be spatially confined to allow selective treatment. The production of reactive oxygen species (ROS) in individual cells results in irreversible damage and cell death [2]. PDT directly initiates apoptotic response in targeted cells [3] without requiring intermediate signal transduction pathways, as is the case in drug-resistant neoplastic cells.

The human pathogen *Staphylococcus aureus* (*S*. *aureus*) bacteria is responsible for a wide range of clinical infections. *S*. *aureus* infection in bloodstream, joints, bones, lungs or heart might lead to death [4]. It is generally present on skin and in the nose of humans. Conventionally treatments involve antibiotics and the drainage of the infected area. Over time the phenomenon of antibacterial resistance has led to the evolution of staphylococcal infections that are no longer responsive to conventional treatments [5]. As such strains have become more and more prevalent. Antibacterial Photodynamic Therapy (aPDT) has emerged as an effective technique for eradication of this pathogen [6].

Various technical aspects of the treatment therapy restrict a higher clinical efficiency of PDT. The absorption spectra of conventionally used PS molecules range within 400–750 nm [7]. However, light has its maximum depth of penetration in tissue within the range of 750–950 nm, termed as the Near-infrared (NIR) Window [7], which has been effectively used for optical imaging in live animals [8]. It is to be expected that a system utilizing this band of the visible spectrum would be more effective in PDT treatments. We have previously demonstrated that higher tissue penetration (6 to 7.4 times greater penetration depth) can be achieved using a femtosecond pulsed NIR (808 nm) laser as compared to a CW 405 nm laser in biological tissue [9]. The experiments further demonstrated that increase in pulse frequency of NIR laser resulted in increasing depth of tissue penetration. The results suggested that by integrating SHG nanoparticles in the PDT process, along with pulsed NIR light, could improve the clinical efficiency of treatments when compared to those involving conventional blue light.

Second harmonic generation (SHG) is a nonlinear optical effect, in which two photons are absorbed by a non-linear crystal, which then emits one photon with double the energy (half the wavelength of the original photons) [10] and is used for a variety of applications in research [11,12]. Being a non–parametric process, SHG can be tuned to achieve localized excitation. With the absence of energy loss involved in SHG, photobleaching is practically avoided [13]. This suggests SHG can be effectively used to generate specific biological responses in targeted applications without affecting surrounding healthy tissue. Recent developments have focused on using SHG to achieve NIR excited PDT in different forms [14].

**Fig 1:**
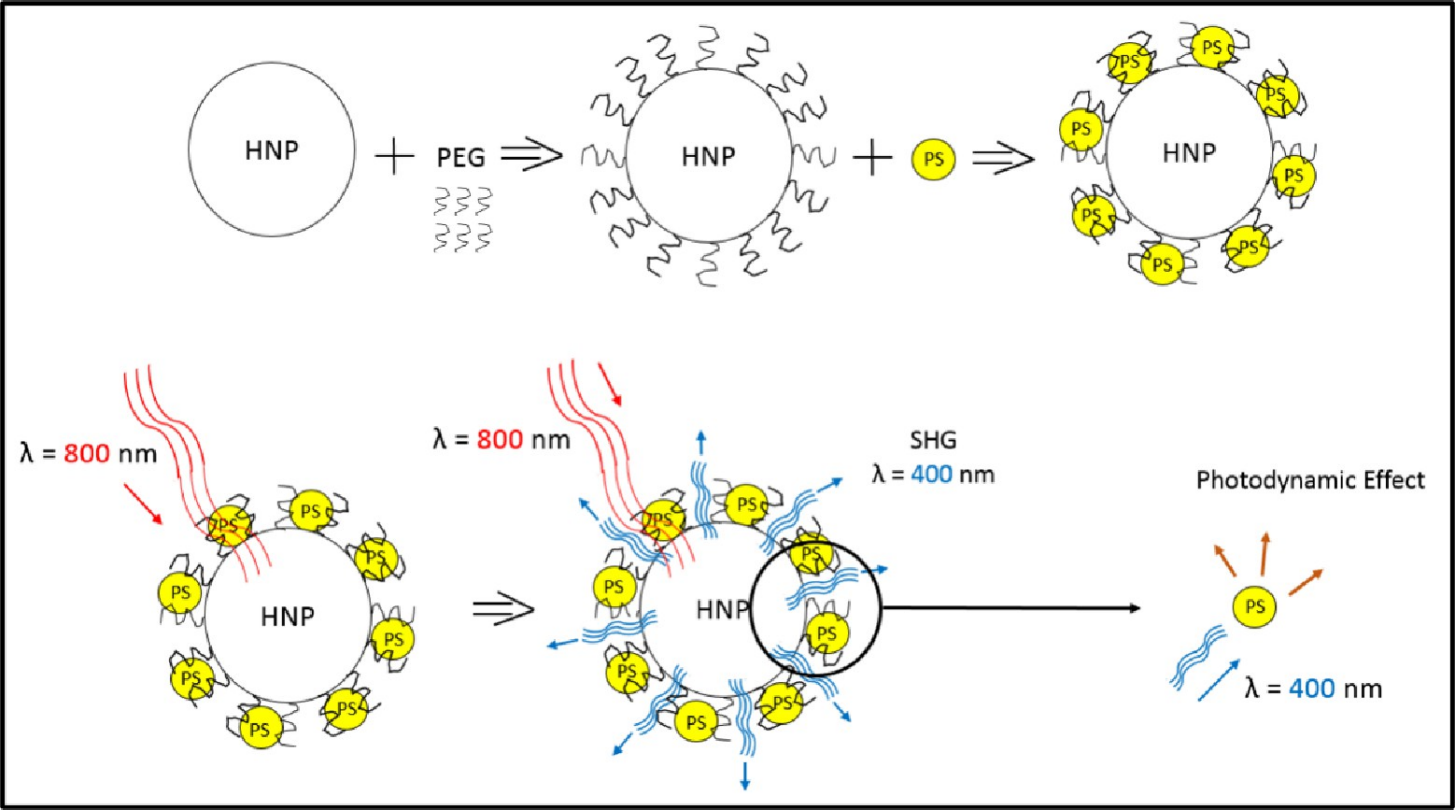
Schematic of photodynamic effect responsive to NIR (Near-infrared) light involving Harmonic Nanoparticle conjugated to Photosensitizer (HNP+PS) using Polyethylene Glycol (PEG).

Barium Titanate (BT), BaTiO_3_, nanoparticles are among the most promising harmonic nanoparticles for SHG enhanced biological applications to have emerged in recent years [15]. Here we synthesize biocompatible Harmonic Nanoparticles (HNPs) conjugated to PS using hydrophobic lipids in order to enhance their targeted localization. We test their photodynamic efficiency by checking the viability of *S*. *aureus* samples after incubation with these conjugates and subsequent irradiation by a NIR 800 nm femtosecond pulsed laser. This irradiation activates the HNPs to emit SHG light that in turn activates the conjugated PS molecules (Fig 1). Activated PS molecules produce ROS thereby killing the targeted cells (photodynamic effect). To demonstrate this novel applicability of photodynamically activating visible light spectrum photosensitizers using NIR light we compared the antibacterial effects of 2 commonly available photosensitizers. Perovskite Barium Titanate (BT) Harmonic Nanoparticles conjugated using Polyethylene Glycol (PEG) to either FDA approved Protoporphyrin IX (PPIX) or Curcumin as PSs are hereafter termed as BT+PPIX or BT+Crcmn respectively.

## 2. Materials and methods

### 2.1 PDT conjugates of nanoparticles and photosensitizers

SHG-PS conjugate nanoparticles were synthesized by mixing and sonicating on ice 6 mg of Barium Titanate (467634, Sigma-Aldrich, USA) with 6 mg of PS—either Protoporphyrin IX (P8293-1G, Sigma-Aldrich, USA) or Curcumin (C1386, Sigma-Aldrich, USA) in 15 ml of 6% Kollisolv® PEG-300 (91462, Sigma-Aldrich, USA) dissolved in distilled water to derive a concentration of 400 μg/ml PDT particles. Subsequently samples were filtered through a 0.22 μm PVDF membrane (FPV-203-013, JetBiofil, China) before use. Samples were imaged after filtration using scanning electron microscope.

### 2.2 Electro-optics

Spectrometry–Spectral Analysis was done using V-730 UV-Visible Spectrophotometer (Jasco, Japan) for UV-vis absorbance and FP-8500 Spectrofluorometer (Jasco, Japan) for fluorescence. Spectra were analyzed using Spectra Manager™ Suite Spectroscopy Software (Jasco, Japan).

Laser Setup–Laser was generated using Millennia® Pro s Series 5SJSPG (Spectra physics, USA) laser setup to generate a 546 nm continuous wave laser which was passed through an ultra-fast oscillator Femtosource® Scientitfic 20 (Spectra Physics, USA) to generate the Near Infrared (800 nm) femtosecond pulsed wave (pulse width 12–15 fs at a frequency 76.9 MHz) beamline. The beamline was appropriately aligned to provide real–time power readings using an ultrafast beam splitter. Power measurements were recorded using a compact power meter PM100D (Thorlabs, Inc.) with S370C thermal power head (Thorlabs, Inc.)

### 2.3 Cell culture

*S*. *aureus* (ATCC 25923) were grown on Brain Heart agar plates (BHA, Acumedia, Lansing, MI, USA) for 24 hours. The cells were then transferred into Brain Heart broth (BH, Acumedia, Lansing, MI, USA) and incubated at 37±1°C with shaking at 170 rpm until reaching an absorbance of A = 0.10 ± 0.02 at 660 nm, corresponding to a final concentration of 10^8^ cells/ml, and diluted appropriately to the final concentration of 10^3^–10^4^ cells/ml with commercially available sterile 0.9% saline solution before the light and dark phototoxicity assays.

### 2.4 Dark toxicity

200 μl bacterial suspensions in 0.9% saline (10^3^–10^4^ cells/ml) in sterile saline solution were incubated with 20 μl of PDT nanoparticle conjugates (400 μg/ml) in the dark with shaking for 30 minutes. Subsequently, aliquots of each sample (100 μl) were spread over BHA plates with a Drigalsky spreader and incubated at 37°C for 24 h. The colony forming units (CFU) were counted using a colony counter Scan 500 (Interscience, Saint-Nom-la-Bretèche, France).

### 2.5 Light phototoxicity

200 μl bacterial suspensions in 0.9% sterile saline solution (10^3^–10^4^ cells/μl) were incubated with 20 μl of PDT nanoparticle conjugates (400 μg/ml) under laser (800 nm, 232–228 mW, 76.9 MHz) with shaking for 10 minutes. Subsequently, aliquots of each sample (100 μl) were spread over BHA plates with a Drigalski spreader and incubated at 37°C for 24 h. The CFU were counted using a colony counter Scan 500.

### 2.6 Scanning Transmission Electron Microscopy (STEM)

The BT harmonic nanoparticles size and microstructure was analyzed by high-resolution scanning transmission electron microscopy (Ultra-High-Resolution Maia 3 FE-SEM, Tescan, Czech Republic) with a STEM (bright and dark) detector and electron beam voltage of 25–30 kV. 10 μl of diluted sample solution was dropped on a grid. An excess of the solution on the grid was removed by filtering paper. The high resolution STEM images were taken mostly at 350 K magnification. Sizes were estimated using FIJI (https://imagej.net/software/fiji/) and particle size distributions generated using a Matlab R2022a (https://www.mathworks.com/products/matlab.html) script developed by us; which we provide for use with any shape and size of nanoparticles (see S1 File).

## 3. Results

### 3.1. Characterisation of Harmonic Nanoparticle–photosensitiser conjugates

**Fig 2:**
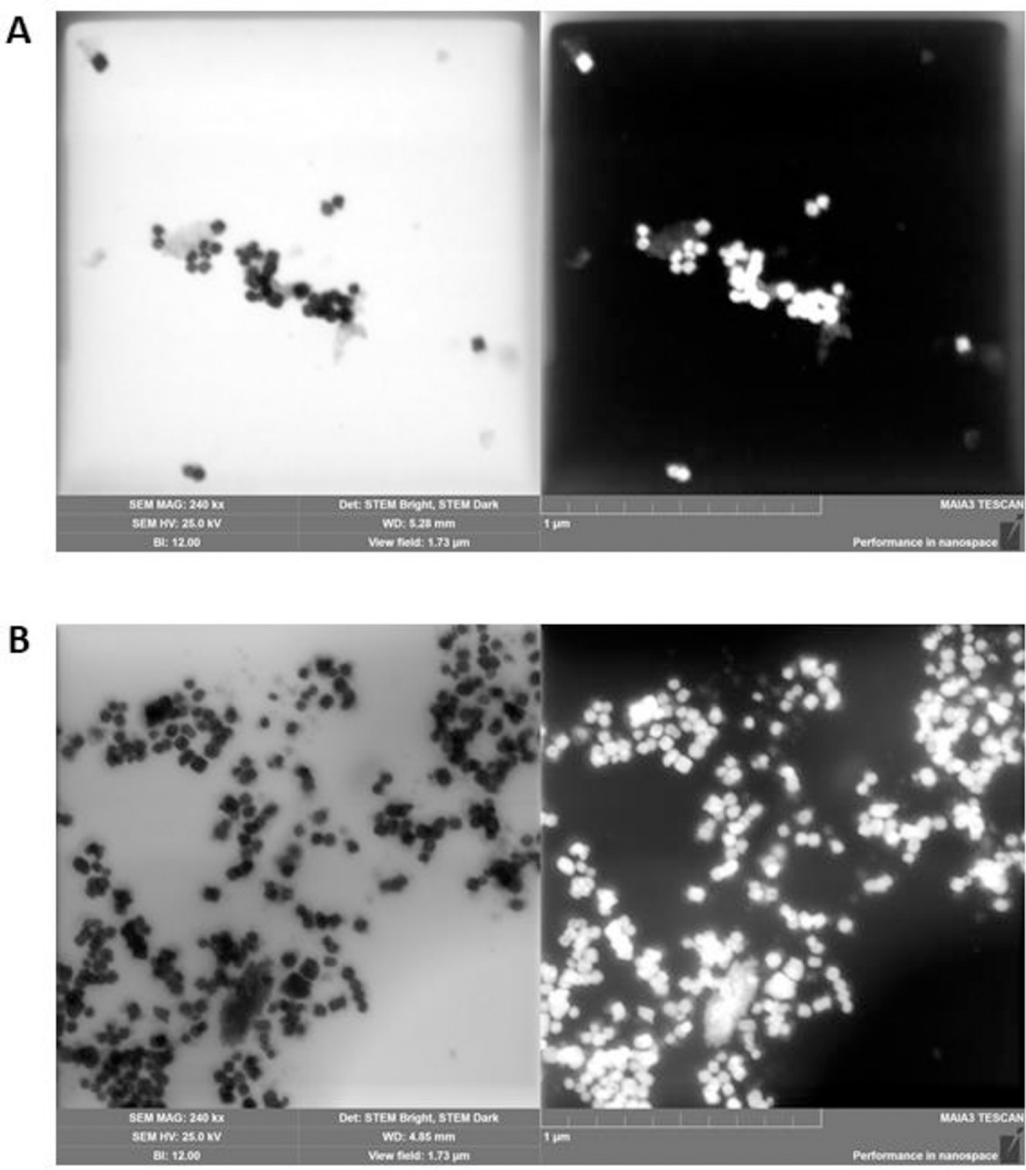
Bright and dark field scanning transmission electron microscope image of Harmonic Nanoparticles of Barium Titanate conjugated with (**A**) Protoporphyrin IX or (**B**) Curcumin after ultrasonication and filtration. Scale Bar– 1 μm.

Barium Titanate HNPs prepared by sonication and filtration for conjugation in our experiments displayed a general size of 53 ± 9nm and 52 ± 9 nm for BT+PPIX and BT+Crcmn respectively (Fig 2) consistent with previous demonstration of 55 nm being the ideal size of Barium Titanate HNPs for SHG efficiency [16].

**Fig 3:**
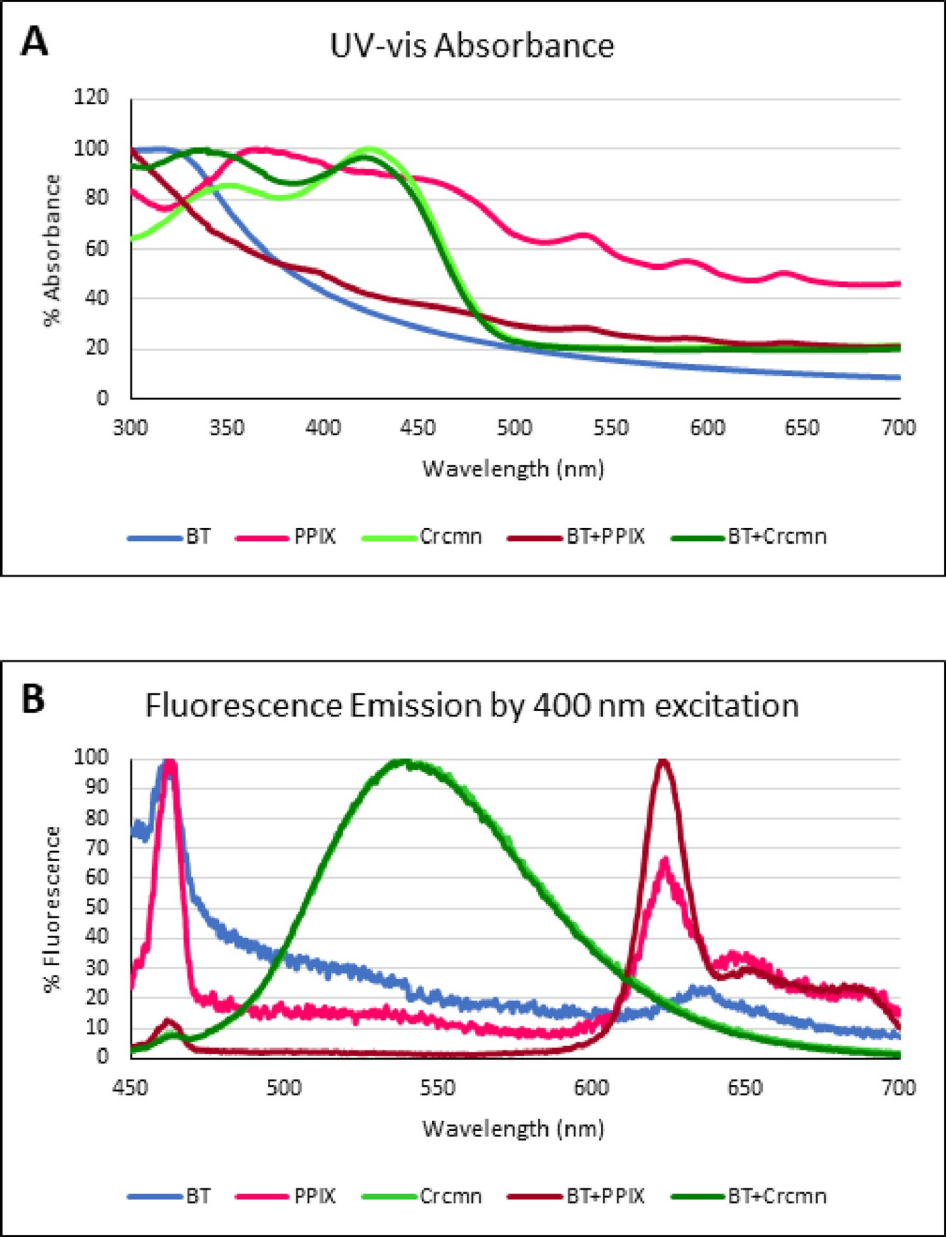
**(A**) Absorbance and (**B**) Fluorescence spectra of BT (Barium Titanate), Protoporphyrin IX (PPIX), Curcumin (Crcmn), BT-PPIX (Barium Titanate conjugated with PPIX) and BT-Crcmn (Barium Titanate conjugated with Curcumin).

Perovskite Barium Titanate (BT) Harmonic Nanoparticles conjugated using Polyethylene Glycol (PEG) to either FDA approved Protoporphyrin IX (PPIX) or Curcumin as PSs were analyzed for their spectral properties (Fig 3A). PPIX by itself showed an absorbance saturation over 369–400 nm with a broad peak at 365 nm. Curcumin by itself showed an absorbance saturation over 415–430 nm with a peak at 424 nm. BT by itself did not show any absorbance in the 350–400 nm range. After conjugation with PPIX the absorbance profile of BT demonstrated a small peak around 400 nm corresponding to that of the photosensitizer PPIX by itself indicating a proximity of particles. Conjugation with Curcumin shifted the absorbance profile of BT demonstrating an additional peak at 340 nm besides the already reported 424 nm peak for Curcumin alone.

BT by itself upon excitation with 400 nm showed a fluorescence emission maximum at 463 nm (Fig 3B). PPIX by itself after excitation with 400 nm showed 2 fluorescence emission maxima at 462 nm and 624 nm (Fig 3B). After conjugation with BT the fluorescence emission profile of PPIX demonstrated a small peak around 463 nm corresponding to that of the photosensitizer PPIX by itself and an enhanced peak at 624 nm indicating a proximity of nanoparticles with the photosensitizer. Curcumin by itself upon excitation with 400 nm showed a fluorescence emission maximum at 540 nm (Fig 3B). Conjugation with BT did not affect the fluorescence emission profile of Curcumin demonstrating a similar peak at 540 nm as was the case for the photosensitizer alone.

### 3.2. Second harmonic generation of Harmonic Nanoparticles and activated emission from conjugated photosensitisers using pulsed NIR irradiation

**Fig 4:**
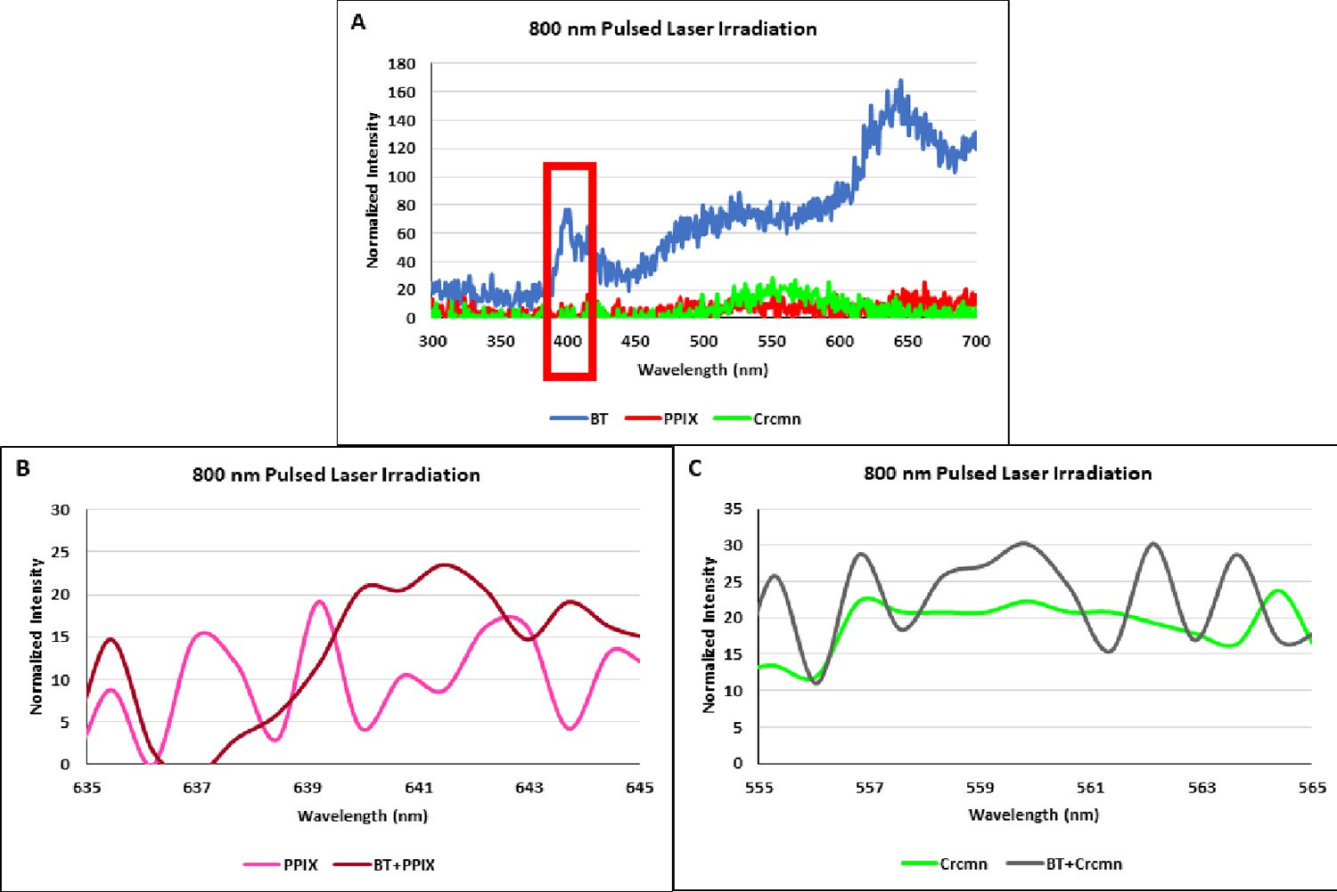
Emission spectra of (**A**) BT (Barium Titanate), Protoporphyrin IX (PPIX), Curcumin (Crcmn); red box highlights the Second Harmonic Generation (**B**) Protoporphyrin IX (PPIX) vs. BT+PPIX (Barium Titanate conjugated with PPIX) and (**C**) Curcumin (Crcmn) vs. BT+Crcmn (Barium Titanate conjugated with Curcumin) after excitation with Near Infrared (800 nm) femtosecond pulsed wave laser beamline.

A femtosecond pulsed laser irradiation at 800 nm on BT by itself showed an emission peak at 400 nm demonstrating the Second Harmonic Generation from harmonic nanoparticles (Fig 4A) while the photosensitizers on their own without conjugation did not show any such response. Conjugation of PPIX with harmonic nanoparticles of BT activated a pronounced emission peak at 641.5 nm which was absent without conjugation (Fig 4B). Similarly, conjugation of Crcmn with harmonic nanoparticles of BT activated an emission peak at 559.8 nm which was absent without conjugation (Fig 4B).

### 3.3. NIR photodynamic effect of HNP-PS conjugates on S. aureus

**Fig 5:**
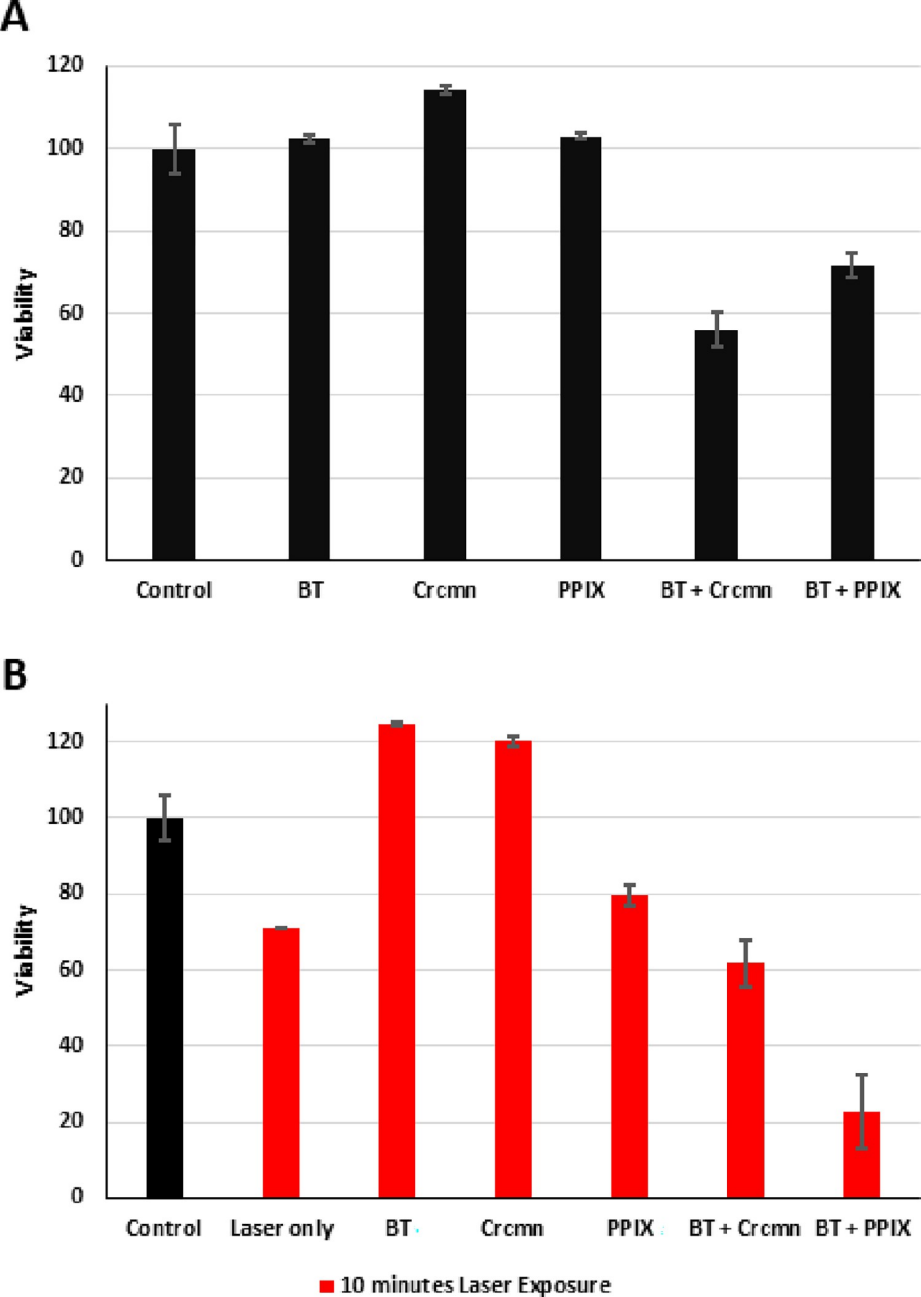
(**A**) Viability of *S*. *aureus* after dark treatment with BT (Barium Titanate) or PPIX (Protoporphyrin IX) or Crcmn (Curcumin) or HNP-PS conjugates. (**B**) Viability of *S*. *aureus* after photodynamic treatment using pulsed NIR laser with either BT+PPIX or BT+Crcmn or HNP and PSs alone normalized to constant power across all irradiated samples with reference to that of laser alone. Cell counts across all labels were kept constant. The error bars indicate standard errors of 6 samples each of HNP-PS conjugates with laser irradiation, 4 samples each of HNP-PS conjugates in dark and 2 samples of each of the rest. Each label represents data for constant cell (200 μl of 10^3^–10^4^ cells/ml) and nanoparticle/photosensitizer (20 μl of 400 μg/ml) concentrations as detailed in methods.

Dark toxicity tests of the prepared HNP-PS conjugates demonstrated that viability of pathogenic *S*. *aureus* after incubation with either BT+PPIX or BT+Crcmn with 30 minutes of dark incubation was reduced by 28.4 ± 3.0% and 44.1 ± 4.2% respectively (Fig 5A) as compared to those of the untreated control. Samples subjected to dark conditions with either BT alone or the PPIX alone didn’t affect the viability significantly as compared to control. However, Crcmn alone showed 14.2 ± 1.1% increase in viability compared to the control.

This indicated that the concentrations of conjugates used without laser irradiation were photodynamically inactive, being unable to generate the half-maximal inhibitory effect on the concentrations of cells involved in the experiment. The results demonstrate the negligible dark toxicity of these Photodynamic conjugates, indicating their biocompatibility for clinical applications.

The photodynamic effect, using 20 μl (concentration 400 μg/ml) of either BT+PPIX or BT+Crcmn with 10 minutes of exposure to pulsed NIR (800 nm) laser radiation reduced the viability of 200 μl (concentration 10^3^–10^4^ cells/ml) of treated *S*. *aureus* samples by 77.3 ± 9.7% and 38.4 ± 6.1% respectively as compared to those of the untreated control (Fig 5B).

Laser exposure alone reduced the viability of treated samples by 28.8 ± 0.1%. BT with laser exposure increased the viability of treated samples by 24.7 ± 0.4%. PPIX alone with 10 minutes laser exposure didn’t have a significant effect on *S*. *aureus* viability, reducing it by 20.4 ± 2.5% similar to that of the laser irradiation alone. Curcumin alone with 10 minutes laser exposure increased the viability of treated samples by 20.2 ± 1.4%. This indicates that the proximity of conjugation to the HNP activates the photosensitizers to generate the desired antibacterial photodynamic effect in the NIR spectral range.

The average power of laser exposure was measured during the course of the experiments (see methods). The readings were normalized to provide a similarity of energy delivery among the samples of nanoparticles and conjugates for the antibacterial photodynamic effect under test.

## 4. Conclusions

### 4.1. Photosensitisers

The activation of photosensitizer Chlorin e6 (absorption bands ∼400 nm and ∼665 nm), by in situ nonlinear optical up-conversion of near-infrared femtosecond pulsed laser radiation (818 nm) using second-harmonic generation in collagen resulted in a fivefold increase in efficiency as compared to two-photon photodynamic therapy alone [17]. However, only fibrillar collagen produces sufficient non-linear up-conversion as compared to monomeric collagen. For effective drug delivery, monomeric small molecules are more useful for effective cellular absorption. But Chlorin e6 has poor water solubility reducing the significance for effective drug delivery in clinical settings.

An artificially synthesized nonlinear optical nano-conveyor named AHU-1 with strong SHG properties conjugated using biocompatible micelles (mPEG–PDLLA) with Chlorin e6 (Ce6) demonstrated improved SHG-mediated PDT [18] using 808 nm light effectively eliminating B16 melanoma cancer cells. But AHU-1 was found to be insoluble in water and readily dissociated, disabling the SHG function. Perovskite BT nanoparticles composite with chemically synthesized Rose Bengal (absorption band ∼ 550 nm) were shown to be able to produce more reactive oxygen species (ROS) *ex vivo* and *in vitro* [14] using 1040 nm light.

These studies indicate that simply coupling SHG capable nanoparticles, stabilized in suspension with conventional PSs, can directly improve the treatment efficacies of PDT without the requirement of new chemical hybrids or syntheses.

### 4.2. Harmonic nanoparticles

Experiments characterizing the SHG by Barium Titanate HNPs demonstrated that within a size range of 70–20 nm, the higher sizes correspond to bulk like properties dominated by volume and the lower sizes correspond to dominant surface plasmon effects both reducing the intensity of the emitted signal [16] with best results demonstrated by particles of 55 nm. The HNPs used in our conjugates were consistent to this size range (Fig 2).

Although BT harmonic nanoparticles are easily synthesized, they require stabilizers in hydrated environments to prevent precipitation. For effective energy transfer to occur upon light activation, the HNP and PS are required to be relatively close. By attaching PEG phospholipids to the nanoparticles, a hydrophobic environment is created surrounding the HNP for drug delivery via lipid-type carriers enhancing the PS delivery [18]. Further, the affinity of PSs for uptake by membrane bound cells and neoplastic tissues increases with an increasing degree of hydrophobicity [19–21]. The PEGylated HNP’s then load PS’s (Fig 1) and enhance efficiently their cellular uptake. PEGylation also improves the colloidal properties of both the HNP and PS [22].

Our experiments indicate that by incorporating appropriate SHG capable BT harmonic nanoparticles into the PDT process, commonly used photosensitizers conventionally responsive only to visible light spectrum (illustratively Protoporphyrin IX and Curcumin respectively) can be made photodynamically reactive to deeper penetrating Near–Infrared light.

### 4.3. Near-Infrared Photodynamic Therapy

Pulsed lasers are advantageous over continuous wave lasers due to the generation of high instant power fluxes and their better energy transfer [23,24]. NIR-induced PDT has been demonstrated to achieve deeper tissue penetration depth as compared to conventional PDT involving visible excitation light significantly improving treatment outcomes [25]. However, this has given rise to two new developments–the requirement of up conversion particles which can transfer the NIR energy into conventional PSs (reactive to visible light) to generate the PDT effect [23,26] and/or synthesis of new PSs which are directly reactive to NIR spectra [27].

The use of up conversion nanoparticles in the NIR-PDT process often requires further synthesis and conjugations of core@shell structures for efficiency of emission [23,26,27]. Instead, we demonstrate that easily prepared harmonic nanoparticles provide a simpler solution. Perovskite nanoparticles like Barium Titanate (BaTiO_3_) provide improved colloidal stability, enhanced cellular uptake efficiency and function as precise SHG sources [28] due to their coherent and non-bleaching signals under femtosecond laser excitation.

We observed that Curcumin alone without light stimulation increased the growth rate of pathogenic *S*. *aureus* by 14.2 ± 1.1% as compared to control. There are reports of the probiotic effect of curcumin when interacting with certain kinds of bacteria. Growth rates of oral bacteria suggested that curcumin can enhance bacterial growth [29] when used without light stimulation involved. The incubation assays with drug for dark phototoxicity were 30 minutes each while with light phototoxicity the assays were concluded within 10 minutes to reduce effect of laser exposure by itself (see methods). Despite these factors the dark toxicity 44.1 ± 4.2% was not significantly different than photo-toxicity 38.4 ± 6.1% in group BT+Crcmn demonstrating that PDT was non-existent in this case. However, for group BT+PPIX the photo-toxicity 77.3 ± 9.7% was significantly higher than dark toxicity 28.4 ± 3.0% demonstrating successful PDT.

In conclusion, our experiments demonstrate that Protoporphyrin IX significantly performs more efficiently than Curcumin for use in SHG mediated Near Infrared Photodynamic therapy of visible light reactive photosensitizers.

Using the novel method described and tested here, PDT from conventional photosensitizers is achieved by incorporating HNPs overcoming the requirement of NIR reactive PSs [30]. Our results demonstrate that any PSs which are responsive to visible spectrum light only [31] can now be used to generate a photodynamic effect under irradiation by deeper tissue penetrating NIR light through incorporation of appropriate HNPs in the SHG process. Conventional techniques to determine Singlet Oxygen yields [18,31] were inapplicable to measure our system as these methods involve assaying spectral absorption of chemical ^1^O_2_ traps; which overlap the photosensitizer excitation spectral bands radiated by SHG from the HNPs in our PDT mechanism.

The alternative method involving steady-state ^1^O_2_ phosphorescence at 1270 nm [32] was investigated and presented a very low signal level. The hardly detectable low signal to noise ratio is accountable to the PEG micelles in our system corroborating with the previously published results [32] where the low signal to noise ratio was due to Triton X-100 micelles being part of the working system.

Recently, Phototheranostics (light activated therapeutic/diagnostics) has attracted great research interest from multidisciplinary research areas with promising applications for modern precision medicine [33]. In this regard, the main focus has been on developing new small organic dyes as photosensitizers which are responsive to the NIR-I and NIR-II biological windows [34] for enhanced optical transmissions.

Our experiments demonstrate an easier alternative by taking photosensitizers which are generally reactive only in the visible spectrum and turning them into NIR light responsive molecules by the intermediation of Second Harmonic Generation emitting nanoparticles which further improve targeted delivery [29]. Adaptation of these processes will improve the clinical efficiencies of photodynamic therapy by overcoming the limitations of low light penetration and targeted cellular drug delivery.

## Supporting information

S1 File Particle count generator program. (DOCX)
