# Supplementary material for: Second harmonic generation nanoparticles enables Near-Infrared Photodynamic Therapy from visible light reactive photosensitizer conjugates
Source: PLoS One. 2022 Sep 29;17(9):e0274954. doi: 10.1371/journal.pone.0274954 (PMC9522301; doi:10.1371/journal.pone.0274954)

Particle Count Generator Program

Second Harmonic Generation nanoparticles enables Near-Infrared Photodynamic Therapy from Visible light reactive photosensitizer conjugates

Ayan Barbora, Fares Yazbak, Svetlana Lyssenko, Vadim Nave, Faina Nakonechny, Paul Ben Ishai and Refael Minnes

Notes on using the Particle Count Generator Program

(ParticleCountGeneratorVersionR2022a.m)

1. Program runs best on Matlab version R2022a and higher.
2. FIJI software (https://imagej.net/software/fiji/) or any other segmentation software may be used to determine length/area covered by each particle after appropriately scaling their Electron Microscope images. The data is saved as a CSV file.
3. The code reads the CSV file. It generates a histogram of counted particles, a normalized distribution, mean size, standard deviation and presents all measured parameters in a text box.
4. The code can be modified to calculate diameters of particles from their areas detected by FIJI software and present a distribution thereof. This requires activation/modification of relevant lines of code described appropriately in Line 10 in the code (as commented in the Matlab script).
5. Likewise, any other shape (square, rectangle, polygon, etc.) can be used for processing by appropriately changing Line 10 in the code (as commented in the Matlab script).
6. The output is saved as a JPG file.

Public Access link -

<https://www.researchgate.net/publication/362580594_ParticleCountGeneratorVersionR2022a>

Illustration of using the code after particle segmentation using FIJI –


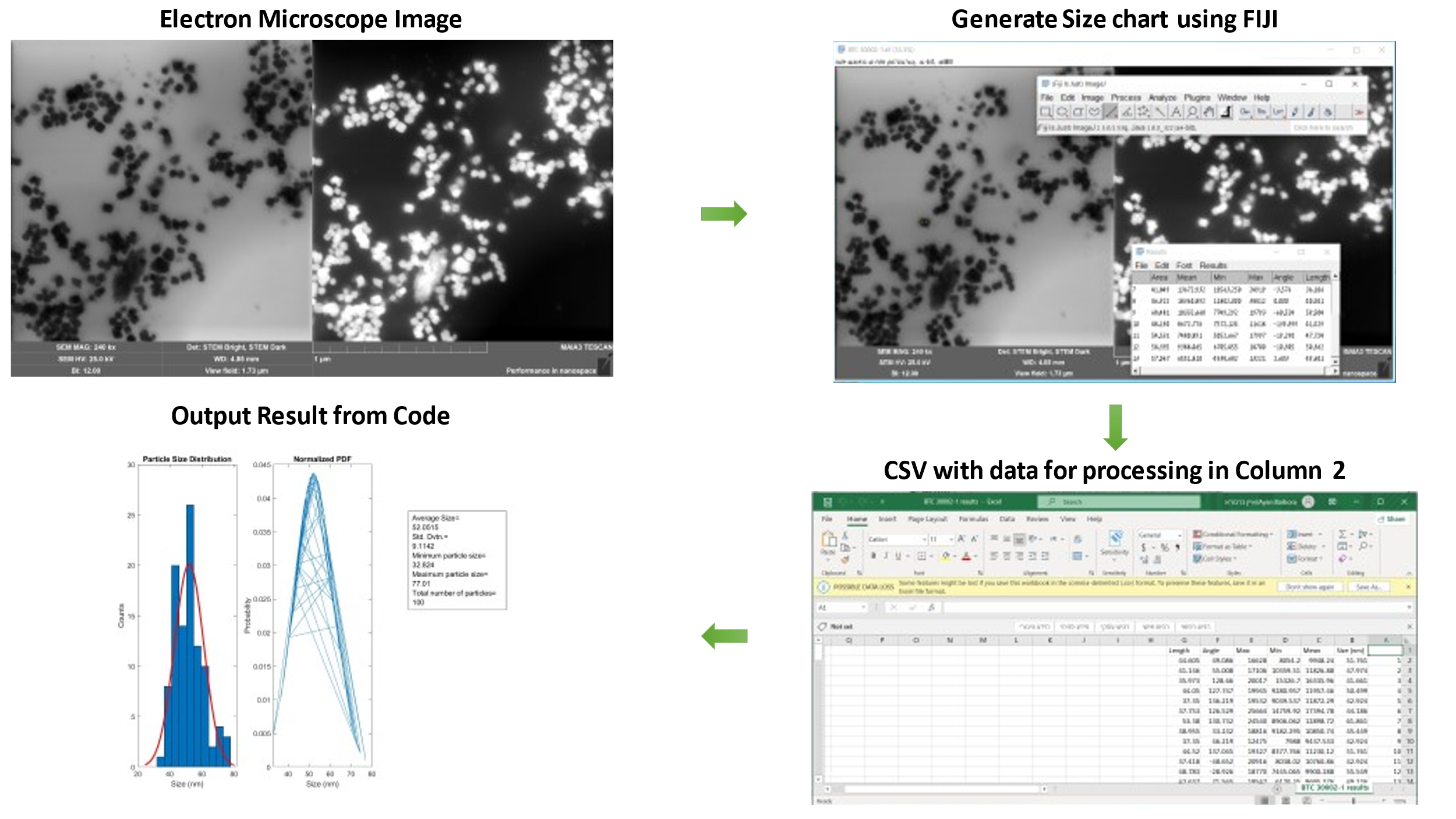

Supplement: S1 File — (DOCX) [file pone.0274954.s001.docx]
